# Alpha-Synuclein Antibody Characterization: Why Semantics Matters

**DOI:** 10.1007/s12035-020-02269-7

**Published:** 2021-01-07

**Authors:** Tiago Fleming Outeiro

**Affiliations:** 1 Department of Experimental Neurodegeneration, Center for Biostructural Imaging of Neurodegeneration, University Medical Center, Göttingen, Germany; 2 Cluster of Excellence “Multiscale Bioimaging: from Molecular Machines to Networks of Excitable Cells” (MBExC), University of Göttingen, Göttingen, Germany; 3 Max Planck Institute for Experimental Medicine, Göttingen, Germany; 4 Translational and Clinical Research Institute, Faculty of Medical Sciences, Newcastle University, Framlington Place, Newcastle Upon Tyne, NE2 4HH UK; 5 Deutsches Zentrum für Neurodegenerative Erkrankungen (DZNE), Göttingen, Germany

**Keywords:** Alpha-synuclein, Neurodegeneration, Protein aggregation, Antibodies, Oligomers

## Abstract

In protein aggregation disorders, we assume that, during the process of protein aggregation, different types of aggregated species (oligomers, protofibrils, fibrils, etc.) are formed, some of which can be toxic to cells/tissues/organs. Recent evidence from numerous studies in cell and animal models of disease suggest that oligomeric species of different proteins might be more toxic that the larger, fibrillar forms. However, we still lack definitive data on the nature of the toxic species, mostly due to our inability to detect and define the various protein species that form as protein aggregate. The terms used are often broad and do not capture inter-laboratory variation in protocols and methods used for the characterization of aggregates. Even antibody-based methods can be ambiguous, as antibodies are delicate tools. Therefore, systematic and interdisciplinary studies are essential in order to guide future developments in the field.

In a study recently published in *Neurobiology of Disease*, Kumar and colleagues conducted an impressive amount of careful and rigorous work on a topic of great relevance in the field of synucleinopathies—the study of different forms and assemblies of alpha-synuclein (aSyn), a protein deeply implicated in these diseases [1].

We were already used to a level of rigor that is characteristic from this research group and, this time, there is no surprise either. The study is systematic, carefully planned and executed, and brings about important knowledge that, if nothing else, highlight the need for uniformization in terms of protocols and language used in the field.

Even if they may be less toxic [2], I find it highly unlikely that large protein aggregates (assumed to be fibrillar in nature) are harmless inside specialized cells like neurons, where trafficking is a vital part of cell physiology. Nevertheless, the idea that soluble oligomeric species of different proteins might be more toxic than the larger, fibrillar forms, is backed up by plethora of laboratory studies [3–5], and by a lack of correlation between the presence of the typical pathological hallmark inclusions and disease (e.g., at autopsy, amyloid-beta plaques and Lewy bodies are often found in the brains of individuals with no overt signs of Alzheimer’s or Parkinson’s disease, respectively). On the other hand, the idea that oligomeric forms of proteins are formed “on pathway” to the formation of the mature amyloid-like fibrils is also supported by numerous in vitro and in vivo studies. However, the field suffers from major limitations: The term “oligomer” is ill-defined, to say the least, as it is often used to refer to a plethora of protein species that is difficult to compare between different laboratories, as this is done according to several different techniques and protocols that gather no consensual guidelines. Therefore, oligomers produced in laboratory A may be, and often are, significantly different from oligomers produced in laboratory B. In fact, we know that even minor changes in the purification and handling of aSyn may lead to the formation of different types of aggregated species and to different pathologies [6, 7].

The Kumar et al. study took brute force and assessed the behavior of a panel of 18 antibodies in the context of in vitro-prepared aSyn species: monomers, oligomers, and fibrils. The authors used a variety of antibody-based techniques, including immunoblot analyses and ELISA, and surface plasmon resonance (SPR), and found that, at the concentrations of aSyn tested, the antibodies lacked the specificity that one would expect based on the literature.

The findings may appear surprising to some but, in fact, I believe they were to be expected, as comparing results obtained in different laboratories is often difficult due protocol differences. For example, the antibodies tested were developed using aSyn species produced in other laboratories, using different methods and, therefore, the results now obtained may not be directly comparable to those previously reported. In addition, and as the authors point out, a major limitation is to know how the aSyn species used as reference in the study relate to those accumulating in the human brain—one may expect them to be very different in fact, due to the absence of the posttranslational modifications that take place in any biological context, and due to absence of protein interactors in an in vitro system. The recent study by Schweighauser et al., using material derived from the brains of individuals with dementia with Lewy bodies or multiple system atrophy, demonstrates the formation of distinct types of assemblies in different diseases [8]. Moreover, the study also demonstrated differences in aSyn assemblies produced in vitro and those found in human brain tissue.

The study by Kumar et al. has merit in as much it highlights the fact that we need to be cautious when assuming the specificity of the numerous antibodies used in the field, including those commercially available, but one should also recognize the antibodies tested have been widely used by many expert groups and shown to be valuable tools. At any rate, perhaps the most striking message of the Kumar et al. study is to highlight the need for more precise guidelines and standardization in the field, so that the community can actually compare results and address important outstanding questions, such as the eternal question of what the toxic protein species is/are. This requires the community to work together, as this is the only way we might move forward and rationally develop tools and strategies for therapeutic intervention in these devastating diseases.

## Data Availability

Not applicable.
